# Application of Continuous Glucose Monitoring for Assessment of Individual Carbohydrate Requirement during Ultramarathon Race

**DOI:** 10.3390/nu12041121

**Published:** 2020-04-17

**Authors:** Kengo Ishihara, Natsuki Uchiyama, Shino Kizaki, Emi Mori, Tsutomu Nonaka, Hiroshi Oneda

**Affiliations:** 1 Department of Food Sciences and Human Nutrition, Faculty of Agriculture, Ryukoku University, Shiga 520-2194, Japan; 2 Department of Life Science, Manchester Metropolitan University, Manchester M1 5GD, UK; 3 Department of Food and Nutrition, Jin-ai Women’s College, Fukui 910-0124, Japan; 4 Tail Ender’s Trail Running Life, Tokyo 176-0004, Japan; 5 Nagatasangyo Co., Ltd, Shiso 671-2544, Japan

**Keywords:** sports nutrition, continuous glucose monitoring, carbohydrate, trail running, Freestyle Libre

## Abstract

Background: The current study intended to evaluate the feasibility of the application of continuous glucose monitoring to guarantee optimal intake of carbohydrate to maintain blood glucose levels during a 160-km ultramarathon race. Methods: Seven ultramarathon runners (four male and three female) took part in the study. The glucose profile was monitored continuously throughout the race, which was divided into 11 segments by timing gates. Running speed in each segment was standardized to the average of the top five finishers for each gender. Food and drink intake during the race were recorded and carbohydrate and energy intake were calculated. Results: Observed glucose levels ranged between 61.9–252.0 mg/dL. Average glucose concentration differed from the start to the end of the race (104 ± 15.0 to 164 ± 30.5 SD mg/dL). The total amount of carbohydrate intake during the race ranged from 0.27 to 1.14 g/kg/h. Glucose concentration positively correlated with running speeds in segments (*P* < 0.005). Energy and carbohydrate intake positively correlated with overall running speed (*P* < 0.01). Conclusion: The present study demonstrates that continuous glucose monitoring could be practical to guarantee optimal carbohydrate intake for each ultramarathon runner.

## 1. Introduction

For the first time in human history, in 2019, Eliud Kipchoge ran the marathon distance in under two hours. Recent advances in the area of sports science significantly contributed to his success. In terms of exercise nutrition, it has been recommended to consume 90 g/h of carbohydrates for endurance exercise [1,2]. This amount has been suggested based on the maximum oxidation of carbohydrate as an energy substrate [3,4] and it is noted that the rate-limiting step to oxidizing this amount of carbohydrate is the gastrointestinal absorption process [1]. 

A longer distance marathon is known as an ultramarathon, and the popularity of these events has increased in recent years [5]. The total energy expenditure of a 160 km ultramarathon reaches about 13000 kcal [6]. Thus, nutritional strategies have to be considered for ultramarathon runners wanting to improve their race results, but also for those focusing primarily on finishing the event. 

GI distress, which is frequently experienced by runners during all types of endurance exercise, makes the current carbohydrate intake recommendation difficult to achieve [7,8,9,10]. Several observation studies have shown that carbohydrate intake during ultramarathon races is lower than the current recommendation for carbohydrate intake. In addition to these statements and recommendations, the optimal nutritional strategies for ultramarathons have been proposed based on a baseline metabolic model [11]. It has been reported that only one study [12] achieved the carbohydrate amount suggested in the current recommendation, while others achieved less than the 60 g/h lower level of the recommendation. The lowest observed average was 31 g/h in slower runners [13]. 

A recently published position statement of the International Society of Sports Nutrition recommended the consumption of 150–400 kcal/h (carbohydrate, 30–50 g/h) [9]. Recent practical recommendations for ultramarathon events offered advice to consume tolerable carbohydrate intake quantities during exercise, which corresponded to 0.8–1.0 g/kg/h of carbohydrate [14]. These values were provided by comparing the race diet between fast and slow runners [13] or by comparing the carbohydrate intake of finishers and non-finishers [12]. 

Optimal nutrition results in a decreased risk of energy depletion, better performance [10], the prevention of acute cognitive decline, and improved athlete safety on ultramarathon courses with technical terrain or those requiring navigation [9]. However, it may prove difficult for the runner to execute the precise nutrition plan [11] and the carbohydrate requirement for ultramarathon racing varies greatly depending on the individual [9]. 

The aim of this study was to evaluate the feasibility of continuous glucose monitoring to improve the carbohydrate intake of ultrarunners using a continuous glucose monitoring system [15,16]. 

## 2. Materials and Methods 

### 2.1. Study Design

This observational study was designed to determine the minimum carbohydrate requirement to maintain blood glucose level and race speed during ultramarathons. All procedures were approved by the Ryukoku University Human Research Ethics Review Board (No. 2016-08-02). All research procedures complied with the code of ethics of the World Medical Association (Declaration of Helsinki). Written informed consent was obtained from all the participants before the commencement of the study. 

### 2.2. Study Population

Seven runners (4 male and 3 female) without injuries volunteered to participate in the study. All the runners had completed 2 to 3 races certified by the International Trail Running Association and the sum of finisher’s points exceeded 12 in the last 3 years, demonstrating their experience in running Ultramarathons. Participant characteristics are presented in Table 1.

### 2.3. Race Course

The present study was conducted during the 2019 Ultra trail Mt. Fuji (https://www.ultratrailmtfuji.com/), held during the last week of April, around Mt. Fuji in Japan (ambient temperature range: 2.3–19.9 °C). The distance of the course covered 165 km and the total elevation was 7942 m. The course included trails, rocks, paths, grasslands, and pavements. The course was divided into 11 segments by 10 timing gates where each runner’s passing time was recorded electronically. Distances between each timing gate were 15 ± 5.4 SD km and varied from 7 to 28 km. Running time and speed between each timing gate were obtained from the official race web site. Running time between each timing gate was 1:58 ± 0:48 and 2:18 ± 0:52 h:m for the top 5 male and female finishers, respectively. All the runners had to run with backpacks to carry necessities, including food, and they could replenish food and fluid at each timing gate. 

### 2.4. Running Speed Data Collection and Standardization

Running speed between each timing gate and overall running speed were obtained from the official race web site. The standard running speed of male and female participants (designated as 100%) for each segment were calculated by averaging the top five male and female finishers, respectively. The running speed of subjects in each segment was standardized using the following formula. The standardized running speed exceeds 100% only when running at a pace comparable to the top 1 and 2 places in each gender:

%Running speed = (The subject’s running speed) / ((Average of top 5 finishers’ running speed in each gender)) × 100, (1)

### 2.5. Glucose Data Collection and Standardization

Blood glucose profile was monitored by a minimally invasive method known as flash glucose monitoring (FGM). Its details have been reported elsewhere [15,17,18]. Briefly, the FGM system (FreeStyle Libre; Abbott Diabetes Care, Alameda, CA) mechanically reads and continuously measures glucose concentration in the interstitial fluid collected from cells immediately below the skin and produces the corresponding ambulatory glucose profile. Subjects were asked to attach the device more than 1 day before the race. The FGM sensor was applied at the back of the upper arm and glucose concentrations were obtained every 15 min [17]. 

**Figure 1:**
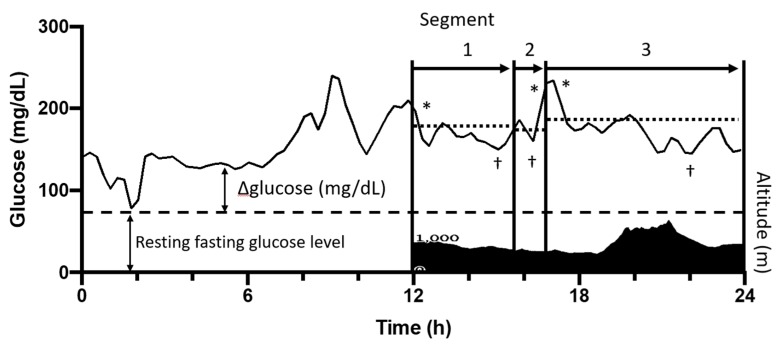
Schematic presentation of the standardization of glucose levels during the race. The overall race course was divided into 11 segments (arrows) by 10 timing gates. The altitude profile of the race course (filled area) and the change of glucose level (solid line) of the first 12 h of the race is shown as a representative result. ΔGlucose level was obtained by subtracting the resting fasting glucose concentration of each runner (dashed line). *, highest value of Δglucose in each segment; †, lowest value of Δglucose in each segment; dotted line, average value of Δglucose in each segment. Running speed (%) was calculated by dividing each runner’s running speed by the average running speed of top 5 finishers.

The glucose concentration of each runner during the race was standardized by subtracting the resting fasting glucose concentration of the runner and was expressed as an increase from resting fasting glucose level (Δglucose). The average, highest, lowest, and the difference between the highest and lowest levels of Δglucose in each segment were used as representative values in each segment (Figure 1). 

### 2.6. Diet Supply Data Collection

Runners were asked to record their entire food and drink intake throughout the race. They reported the timing and volume of consumed food products and fluids based on pictures taken throughout the race. Food products and fluids consumed more than 60 min before the race start were not included in the calculation of nutritional intake. The energy and carbohydrate intake during the race were calculated based on the nutrition information provided by manufacturers. If data was not available, intakes were calculated based on the standard tables of food composition in Japan 2015 - (7th revised edition) [19]. The energy and carbohydrate intake were expressed relative to kg of pre-race body weight, per hour of running time. All foods were categorized with reference to previous research [20] as: sports drinks (isotonic and hypertonic formulas), gels, cola, other fluids (all other drinks consumed), sweets, fruits, bars, noodles, bread, rice products and other solids (all other products consumed). 

### 2.7. Statistics

The data reported in the text, tables, and figures are presented as means and standard deviations, unless otherwise specified. Data were processed and analyzed in GraphPad Prism for Mac (version 8.3.1, GraphPad Inc., San Diego, CA, USA). Pearson’s correlation coefficients were used to investigate the associations between running speed, glucose level, and carbohydrate intake. One-way ANOVA followed by Tukey’s post-hoc test were used to compare the differences between each runner’s blood glucose level. Results were considered significant when *P* < 0.05. 

## 3. Results

### 3.1. General Results

The running speed of the participants ranged from 3.90 to 7.22 km/h with a standardized running speed ranging from 49.0% to 90.1%. 

### 3.2. Relationship between Glucose Level and Running Speed

All participants were within the expected normoglycemic range during exercise (72–252 mg/dL) with the exception of one participant who exhibited a lowest value of 61.9 mg/dL as shown in Table 2. Carbohydrate mainly supplied total energy intake during the race (77.6 ± 8.58SD% of total energy intake). 

Each runner consumed carbohydrates from liquids, gels, fruits, sweets or solids as shown in Table 3. Six of 7 runners consumed more than 55% of their carbohydrates from liquids and gels (55.3% to 74.8%) except for one runner (28.4%, subject 3). Carbohydrate intake from solids ranged from 21.1% to 42.8% in the six runners and 63.8% in the other runner, who showed the highest fat intake among 7 runners (subject 3). 

**Figure 2:**
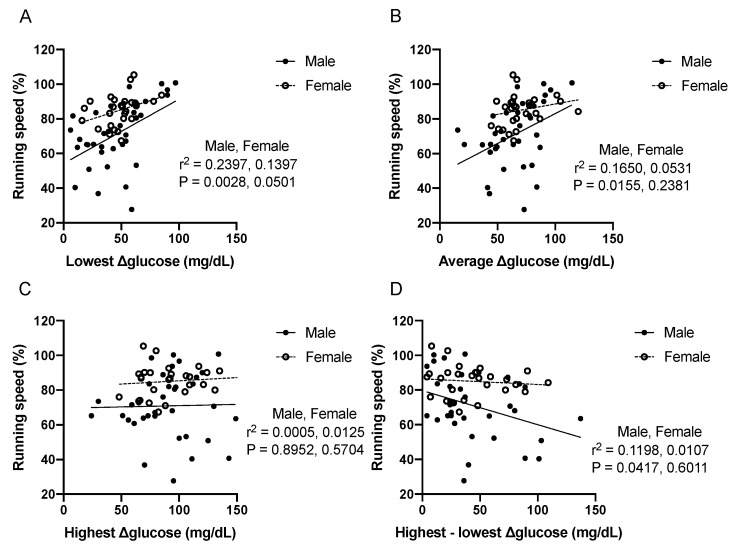
Scatter plots showing relationships between glucose level and running speed. The lowest (**A**), average (**B**), highest (**C**), and difference between highest and lowest (**D**) value of Δglucose levels were calculated as described in Figure 1. Each plot indicates one segment.

The average, highest, lowest, and the difference between the highest and lowest levels of Δglucose in 11 segments were subjected to correlation analysis between running speed and blood glucose level. Figure 2 shows the relationship between glucose level and running speeds in each segment. The lowest (r^2^ = 0.2397, *P* = 0.0028; r^2^ = 0.1397, *P* = 0.0501 for male and female, respectively) and average (r^2^ = 0.1650, *P* = 0.0155; r^2^ = 0.0531, *P* = 0.2381 for male and female, respectively) levels of Δglucose had a significant positive correlation with running speed, but not for the highest levels of Δglucose (r^2^ = 0.0005, *P* = 0.8952; r^2^ = 0.0125, *P* = 0.5704 for male and female, respectively) in male runners. Similar but not significant tendencies were observed in female runners. Interestingly, a significant inverse correlation (r^2^ = 0.1198, *P* = 0.0417; r^2^ = 0.0107, *P* = 0.6011 for male and female, respectively) was observed between running speed and the difference between highest and lowest (D) in male runners. 

### 3.3. Relationship between Energy and Carbohydrate Intake and Running Speed

**Figure 3:**
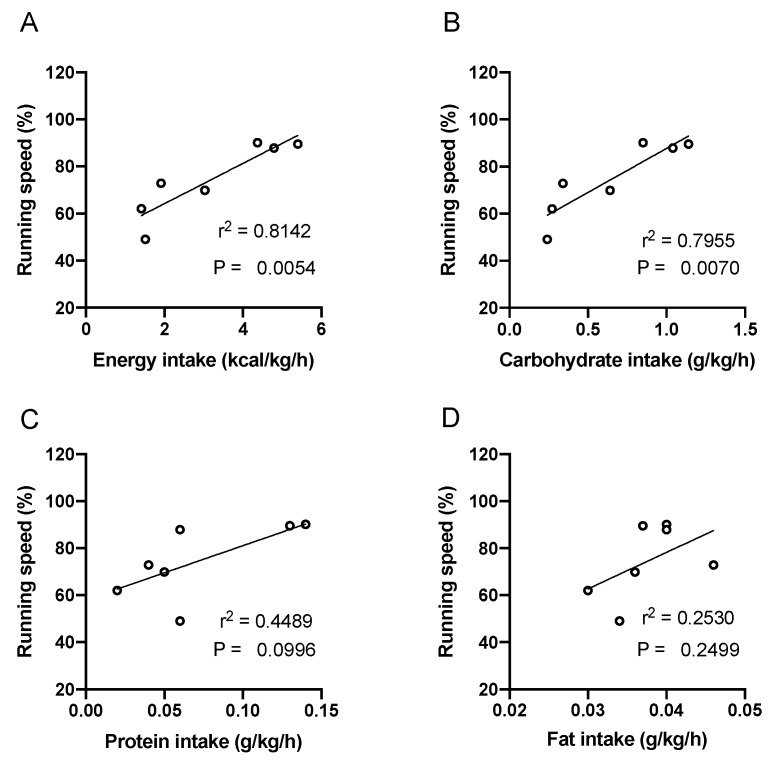
Scatter plots showing relationships between nutrient intake and running speed. The intake of energy (**A**), carbohydrate (**B**), protein (**C**), and fat (**D**) were calculated based on consumed food products and fluids. Each plot indicates one runner.

Energy intake exhibited a significant positive correlation with running speed (r^2^ = 0.8142, *P* = 0.0054). Energy intake ranged from 1.41 to 5.40 kcal/kg/h, which is the equivalent of 86.2 to 226.7 kcal/h. A significant correlation was also found between carbohydrate intake and running speed (r^2^ = 0.7955, *P* = 0.0070). Carbohydrate intake ranged from 0.27 to 1.14 g/h/kg (1.1 to 4.6 kcal/h/kg), which is the equivalent of 16.3 to 52.9 g/h. The energy intake from carbohydrates contributed 63% to 87% of the total energy consumed during the race. No significant correlations were observed between running speed and energy intake from protein and fat (Figure 3). 

### 3.4. Relationship between the Amount of Carbohydrate Intake and Maintenance of Glucose Level during Race

**Figure 4:**
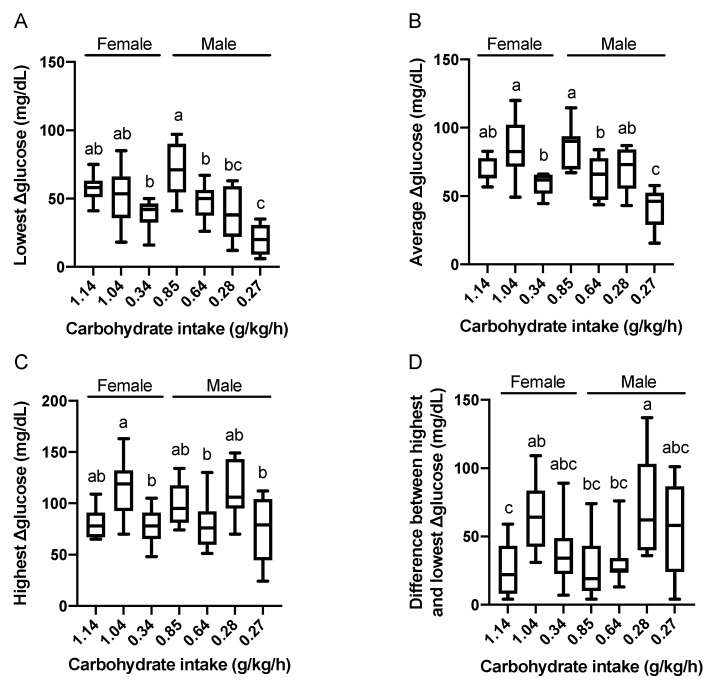
Relationships between carbohydrate intake and the lowest (**A**), average (**B**), highest (**C**), and the difference between the highest and lowest (**D**) value of Δglucose levels. Carbohydrate intake of each runner is expressed in the X-axis. Data are expressed using box-and-whisker plots to indicate the minimum, first quartile, median, third quartile, and maximum. Bar height indicates the average of the dots. Values without common superscript are significantly different, *P* < 0.05

Carbohydrate intake of the seven participants varied within the range of 0.27 to 1.14 g/kg/h and the carbohydrate intake of four subjects (0.27, 0.28, 0.34, and 0.64 g/kg/h) were less than the recently published practical recommendations for ultramarathons. The lowest Δglucose levels of the four subjects were 55.5%, 27.2%, 54.3%, and 66.9% compared to that of the subject who consumed 0.85 g/kg/h, respectively (*P* < 0.05). Likewise, the average level of Δglucose of the four subjects were 48.2%, 68.6%, and 73.6% compared to that of the subject who consumed 0.85 g/kg/h, respectively (*P* < 0.05). Runners who consumed 1.04 or 0.28 g/kg/h of carbohydrate showed higher values in the highest Δ glucose levels and the difference between the highest and lowest blood glucose among seven runners, which seemed to be their specific characteristics (*P* < 0.05, Figure 4). 

## 4. Discussion

The aim of this study was to evaluate the feasibility of continuous glucose monitoring to improve the carbohydrate intake [9,13] of ultrarunners using a continuous glucose monitoring system. Overall carbohydrate intake in three of seven subjects were far below the recommended carbohydrate intake (30–50 g/h or 0.8 g/kg/h). A significant positive relationship was observed between higher carbohydrate intake and faster running speed as was expected from the results of previous studies [12,13]. The present study demonstrates that the avoidance of relatively low blood glucose concentrations, achieved through the intake of sufficient carbohydrates, impaired running speed during the ultramarathon. Conversely, there was no association between the highest blood glucose concentrations obtained with running speed, indicating that control of glucose homeostasis, rather than the rapid availability of carbohydrates, is the key determinant of performance. Runners consuming less than 0.8 g/kg/h of carbohydrates tended to have a reduced running speed associated with a result of low blood glucose. 

Carbohydrate intake of 30–60 g/h is an established recommendation for endurance sports, with even higher amounts (i.e., up to 90 g/h and a glucose:fructose ratio of 2:1) being advocated for exercise bouts lasting more than 3 h [1,2]. However, there is a disparity between this recommendation and actual intakes in ultramarathon runners. Observation studies have demonstrated that actual carbohydrate intake during ultramarathons is less than 60 g/h in most runners [6,13,21], including slower runners consuming 37 g/h [14], with very few runners taking more than 60g of carbohydrates [22,23]. There are numerous barriers to achieve consumption of 90 g/h of a multiple-transportable carbohydrate blend. First, the absolute exercise intensity of an ultramarathon is not as high as some other endurance activities because of its extremely long duration (6, 13, 24, 48, 72 h, 6 or 10 days) [24]. Secondly, the rate-limiting step for oxidizing 90 g of carbohydrate per hour is intestinal absorption which may be affected by undertaking exercise of this intensity and duration due to changes in splanchnic blood flow. In addition, ultramarathon runners lose appetite as a result of heat, endotoxin, or vertical shaking of their digestive system during rough terrain races [24,25,26]. Thirdly, a practical limitation is that ultramarathon runners have to carry their food and fluid in their backpacks during long hours of racing, resulting in an increase in exercise intensity due to the additional weight being carried [14]. Fourthly, runners may have physical difficulties in consuming foods when they are keeping balance with both hands when running down steep mountains or climbing steep slopes. 

For these reasons, discrepancies easily occur between the recommended amount and the actual amount of carbohydrate intake. However, the optimal amount of carbohydrate varies greatly depending on the individual [9]. Therefore, the application of a continuous glucose monitoring system could be a practical and fast method to estimate optimal carbohydrate intake for each runner. 

Given the duration typical of ultramarathons (6 to 48 h), it is not feasible to meet carbohydrate consumption in its entirety during a race. Energy deficiency is common in ultramarathons [8,9,10,12,13,20,21,27]. Several studies using a doubly labeled water technique or respiratory gas analysis have estimated that energy expenditure during ultramarathons is about 13000 kcal [6,28,29]. The amount of carbohydrates consumed during a 160 km ultramarathon can be speculated from indirect calorimetry. The respiratory exchange ratio was 0.91 during the first 64.5km of the 160km race [29] and was 0.85 immediately after the 330km race [30]. Therefore, carbohydrate oxidation likely provided 50.0%–68.3% of energy expenditure, which is equal to 6500–9100 kcal (1625–2275 g) in the 160 km race. 

Gluconeogenesis and hepatic glycogenolysis play an important role to maintain blood glucose levels during prolonged exercise in a fasted or carbohydrate deficient status. Previous studies have reported rates of gluconeogenesis and hepatic glycogenolysis as 0.07 g/kg/h and 0.03 g/kg/h, respectively, in a resting state in low carbohydrate-fed subjects [31]. The sum of these two values (0.1 g/kg/h), endogenous glucose production, would be the minimum amount of carbohydrate required to maintain blood glucose during a resting state. The endogenous glucose production significantly increases to 0.36 g/kg/h during exercise at 55% of peak power output [31] or to 0.48 g/kg/h during exercise at the lactate threshold level in fasted, well trained subjects [32]. Consistently with these findings, three subjects in the present study with a carbohydrate intake of less than 0.48 g/kg/h could not maintain their blood glucose concentrations during the ultramarathon race. 

The main limitation of this study is the small number of participants. The present study supports the effectiveness of a recently published position statement of the International Society of Sports Nutrition [10] and practical recommendation for ultramarathon participants to prevent hypoglycemia during exercise. Relationships among carbohydrate intake, the lowest ∆glucose, and running speed are relevant in male runners rather than female runners. These observations coincide with the previously reported gender-specific differences in fuel utilization during exercise. Women showed higher lipid oxidation caused by higher plasma adiponectin [33], higher muscle triglyceride utilization [34], low plasma glucose [35], and higher fasting hepatic glucose uptake [36] compared to men. However, more subjects are required to conclude that the observed differences between male and female runners were derived from gender-specific factors. 

Hydration and GI distress are negligible factors affecting running speed. Hydration is a factor causing GI distress [37], but these factors could not be standardized in the study. Dehydration issues were not observed, which may be associated a steady rain during the race. These two factors should be quantitatively assessed and statistically analyzed as a factor affecting running speed in larger numbers of participants. 

The insufficient standardization of food intake before and during the race is another limitation. The following factors should be appropriately controlled in future research: pre-race meals within 48 h of the start of the race, caffeine intake, gastrointestinal distress, and objective recording of food and drink intake by action cameras as reported [20]. 

Another limitation of this study is a slower rise and generally lower glucose peak values in the FGM system used in the present study as compared with the blood sampling, and this may underestimate the effect of carbohydrate ingestion on glucose response [18]. Nevertheless, the non-invasive and fast understanding of fluctuations of glucose level according to the specific characteristics of each athlete would be useful to plan and modify a personal nutrient strategy during an ultramarathon race. 

The other limitation of the present study was large fluctuations in running speed in the ultramarathon. The running speeds in 11 segments varied in a range of 5.5 to 14.3 km/h and 4.8 to 11.8 km/h even in top five male and female runners, respectively. We speculated that these fluctuations in running speed were mainly associated with two factors: terrain [26] and physiological changes such as muscle fatigue and energy deficiency. Therefore, the running speeds of the subjects were standardized using the top 5 finishers to explore the relationship between blood glucose levels and running speed. The precise and objective power meters for running, which are already applicable in cycling studies [38], or accurate physical workload calculation based on GPS monitoring, would enable more accurate analysis between running performance and blood glucose. 

## 5. Conclusions

In conclusion, the present study demonstrates that continuous glucose monitoring could be practical to guarantee optimal carbohydrate intake for each ultramarathon runner. Decreases in blood glucose during ultramarathons may be attributed to many factors, including sub-optimal carbohydrate intake. Additionally, individual characteristics such as the sex, age, or energy intake of each runner may have had a greater influence on blood glucose fluctuations across the race; thus, utilizing a continuous glucose monitor may help inform better race nutrition strategies. 

## Figures and Tables

**Table 1:** Clinical characteristics of male and female subjects.

	Male	Female	*P*
Age (year)	41.5 ± 6.2	42.6 ± 1.2	0.627
Height (cm)	172.9 ± 2.7	158.0 ± 6.5	0.019
Weight (kg)	66.0 ± 9.3	47.9 ± 3.8	0.036
BMI (kg/m^2^)	22.2 ± 2.8	18.9 ± 0.7	0.116
Lean body mass (kg)	56.3 ± 5.9	40.7 ± 4.3	0.012
Fat mass (kg)	22.2 ± 2.8	18.9 ± 0.7	0.116

Values are means ± SD (male, *n* = 4; female, *n* = 3).

**Table 2:** The total energy and nutrient intake, and glucose concentration during the ultramarathon.

	Subject	FS	1	2	3	MS	4	5	6	7
Sex		F	F	F	M	M	M	M	M	
Running speed	(%)	100	89.5	87.9	72.9	100	90.1	70.0	62.0	49.0
	(min/km)	6.37	5.70	5.60	4.64	8.01	7.22	5.60	4.96	3.90
Energy intake (kcal/kg/h)		-	5.40	4.79	1.91	-	4.37	3.03	1.41	1.46
Carbohydrate intake (g/kg/h)		-	1.14	1.04	0.34	-	0.85	0.64	0.27	0.28
Protein intake (g/kg/h)		-	0.132	0.061	0.042	-	0.143	0.051	0.021	0.021
Fat intake (g/kg/h)		-	0.037	0.040	0.046	-	0.040	0.036	0.030	0.029
Glucose (mg/dL)										
During race	Average	-	131	137	104	-	145	134	121	164
	SD	-	11.9	30.2	15.0	-	20.4	20.2	22.9	30.5
	Highest	-	173	224	151	-	193	198	189	240
	Lowest	-	105	79	62	-	100	94	83	103
Resting fasting		-	53	58	40	-	57	68	83	98

FS and MS, female and male standard running speed, which correspond to the average of the top 5 finishers in each sex. F, female; M, male.

**Table 3:** Carbohydrates consumed per product type (g/kg/h).

Subject	1	2	3	4	5	6	7	% of Total
**Liquids and gels**	**0.85**	**0.71**	**0.10**	**0.57**	**0.35**	**0.16**	**0.16**	**58. 8 ± 15.1**
Sports drink	0.34	0.05	0.04	0.01	0.00	0.05	0.04	11.8 ± 10.8
Cola	0.00	0.08	0.00	0.05	0.03	0.06	0.02	6.9 ± 7.5
Gel	0.51	0.57	0.04	0.51	0.32	0.05	0.06	37.2±19.6
Other liquid	0.00	0.01	0.01	0.01	0.00	0.01	0.03	3.0 ± 4.3
**Fruits and sweets**	**0.05**	**0.06**	**0.03**	**0.01**	**0.06**	**0.00**	**0.00**	**4.2 ± 3.7**
Fruit	0.04	0.04	0.02	0.01	0.06	0.00	0.00	3.8 ± 3.5
Sweet	0.01	0.02	0.00	0.00	0.00	0.00	0.00	0.4 ± 0.7
**Solids**	**0.24**	**0.27**	**0.21**	**0.27**	**0.23**	**0.10**	**0.12**	**37.0 ± 13.9**
Bar	0.05	0.00	0.00	0.05	0.00	0.00	0.00	1.6 ± 2.7
Noodle	0.02	0.02	0.08	0.00	0.00	0.01	0.00	4.2 ± 8.1
Bread	0.00	0.05	0.05	0.01	0.04	0.04	0.03	7.9 ± 6.5
Rice product	0.00	0.19	0.09	0.18	0.15	0.04	0.08	18.9 ± 9.7
Other solid	0.17	0.01	0.00	0.04	0.03	0.01	0.00	4.4 ± 4.9
**Total**	**1.14**	**1.04**	**0.34**	**0.85**	**0.64**	**0.27**	**0.28**	

Subject numbers are identical to Table 2. The subtotal of each category is shown in bold.
